# A Technical Nuance to Avoid Lumbar Five Radiculopathy with Anterior Lumbar Fusion and Posterior Instrumentation

**DOI:** 10.1155/2021/5514720

**Published:** 2021-03-24

**Authors:** Matthew T. Neal, Maziyar A. Kalani, Mark K. Lyons

**Affiliations:** Department of Neurologic Surgery, Mayo Clinic College of Medicine and Science, 5777 East Mayo Boulevard, Phoenix, AZ 85054, USA

## Abstract

The anterior lumbar interbody fusion (ALIF) is a well-established procedure used to treat a multitude of spinal pathologies. When performed at the L5-S1 level, the ALIF is often supplemented with posterior pedicle screw and rod fixation. Because the interbody device can restore disk and foraminal height, one benefit of the ALIF procedure is indirect neural decompression in the spinal canal and neural foramina. If the contour of the posterior rod is not matched to the exact position of the tulip heads on the pedicle screws, spondylolisthesis can be introduced, leading to foraminal stenosis and nerve compression. This concern is particularly germane when the posterior instrumentation is placed percutaneously without any direct foraminal decompression. In this report, we describe a patient who had an L4-S1 ALIF, resulting in new L5-S1 retrolisthesis and worsening L5 radiculopathy. Technical nuances and avoidance strategies are discussed.

## 1. Introduction

The anterior lumbar interbody fusion (ALIF) is a popular procedure and is being performed by spinal surgeons at an increasing rate [1]. The procedure can be used to treat diverse pathologies and has many benefits. Demonstrated radiographic advantages include effective restoration of disk height, reduction of spondylolisthesis, and improved spinopelvic parameters [2, 3]. The approach allows for a complete discectomy and placement of a large interbody cage, resulting in solid anterior column support and fusion rates in excess of 90% [4]. The ALIF has also been associated with improved patient-reported outcome measures [2, 5].

Posterior instrumentation is commonly added to augment the ALIF. Posterior instrumentation is particularly helpful in cases with high sacral slope or isthmic spondylolisthesis to avoid instrumentation failure or pseudoarthrosis [6]. To minimize devascularization of paraspinal musculature, delayed muscle atrophy, disruption of muscular insertion to the spinous processes, and other approach-related injury, ALIF is often combined with percutaneous rod and screw fixation inserted through paramedian incisions [7, 8].

The ALIF procedure has been associated with diverse complications such as vascular injuries, injuries to other structures in the peritoneal compartment, fractures, pseudoarthrosis, and neural injury [9–13]. At the L5-S1 level, the ALIF has been associated with L5 radiculopathies [14, 15]. Most commonly, L5 radiculopathy has been contributed to a stretch neuropraxia.

In this report, we discuss another source of postoperative L5 radiculopathy following L5-S1 ALIF with posterior instrumentation. We describe a scenario where preexisting L5-S1 foraminal stenosis was exacerbated when the rods were inserted and retrolisthesis was introduced. Strategies to avoid this complication are discussed.

## 2. Case Presentation

The 61-year-old male patient presented with chronic low back pain and progressive bilateral thigh pain following both lumbar 4 and 5 dermatomal distributions. He also described subjective bilateral lower extremity weakness and numbness in the left foot. He experienced a progressive functional decline, despite extensive conservative treatment modalities.

**Figure 1:**
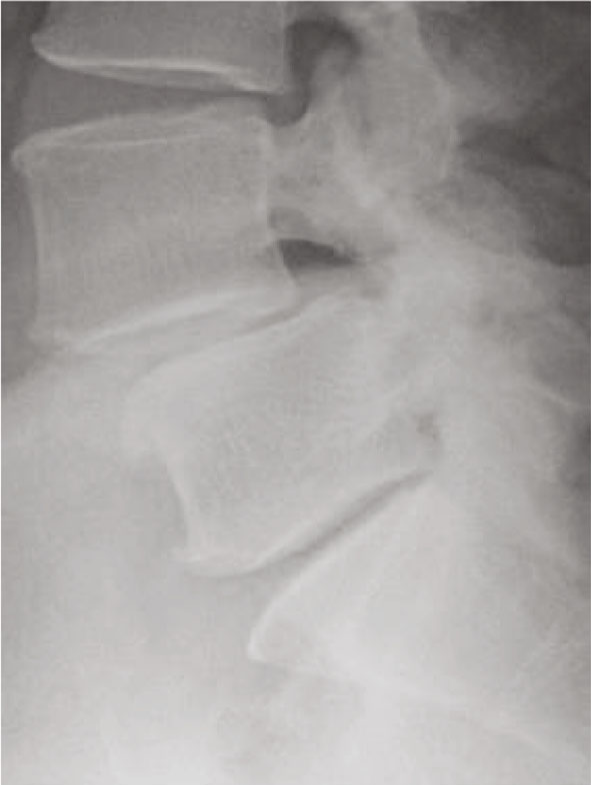
Preoperative lateral lumbar X-ray.

**Figure 2:**
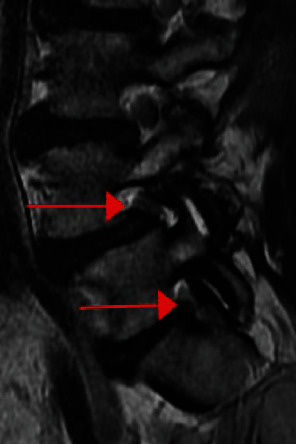
Preoperative T2 sagittal MRI demonstrating spondylolisthesis at L4-5 and foraminal stenosis at both L4-5 and L5-S1.

X-ray and magnetic resonance imaging (MRI) demonstrated grade 1 spondylolisthesis at L4-5 and bilateral foraminal stenosis at both L4-5 and L5-S1 (Figures 1 and 2). The patient elected to pursue anterior lumbar fusions at L4-5 and L5-S1, followed by posterior percutaneous L4-S1 pedicle screw and rod fixation.

**Figure 3:**
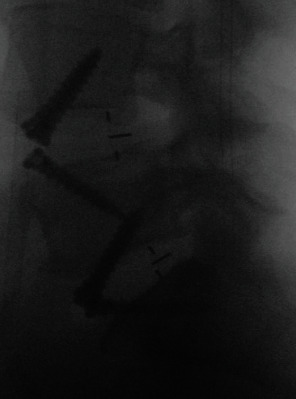
Intraoperative sagittal X-ray demonstrating anterior interbody grafts.

After a standard retroperitoneal exposure of the L4-5 and L5-S1 disk spaces, we performed meticuluous discectomies. We used disk space trials to determine the optimal size for the interbody cages. The interbody cages (Sovereign™ Spinal System, Medtronic Sofamor Danek USA, Inc., Memphis, TN) containing demineralized bone matrix were inserted at both levels and secured with integrated screws. X-rays (Figure 3) demonstrated good restoration of disk height at both levels. Anterolisthesis was reduced at the L4-5 level, and neutral sagittal alignment was maintained at L5-S1.

The patient was positioned prone for placement of posterior instrumentation. Using the StealthStation® S8® Navigation System, pedicle screws (CD Horizon™ Solera™ Voyager™ Awl-Tap Screws, Medtronic Sofamor Danek USA, Inc., Memphis, TN) were inserted bilaterally through paramedian incisions. Screw insertion was uncomplicated and supplemented by free-run electromyography (EMG) monitoring. Satisfactory positioning of the pedicle screws was confirmed intraoperatively using the O-arm™ imaging system (Medtronic, Minneapolis, MN).

**Figure 4:**
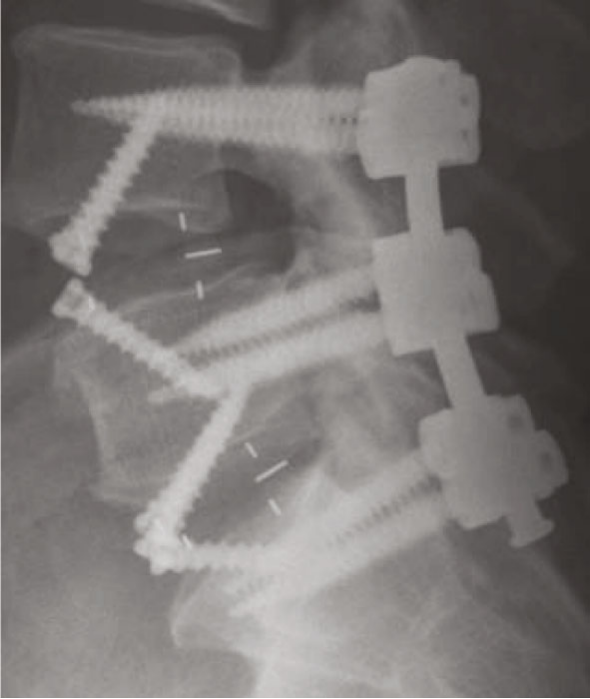
Postoperative lateral X-ray.

Rods spanning from L4-S1 and set screws were then inserted utilizing the threaded, extended tabs attached to the tulip heads of the pedicle screws. The set screws were tightened with the appropriate torque, and the extended tabs were removed. Postoperative X-rays (Figure 4) were obtained.

**Figure 5:**
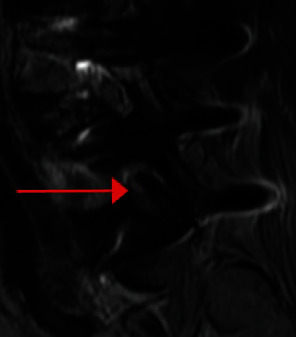
Postoperative sagittal T2 MRI demonstrating persistent L5 nerve compression in the L5-S1 foramen (arrow).

**Figure 6:**
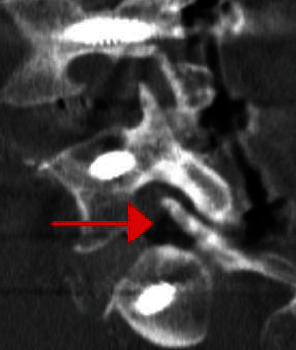
Postoperative sagittal CT scan demonstrating persistent L5 nerve root compression in the L5-S1 foramen (arrow).

Immediately following surgery, the patient experienced increased right-sided gluteal pain which was treated with a short course of oral steroids and gabinoids. However, at his second postoperative visit, 4 weeks after surgery, he had developed 4/5 weakness with right ankle dorsiflexion. A postoperative CT confirmed satisfactory positioning of the instrumentation. The postoperative MRI (Figure 5) and CT scan (Figure 6) demonstrated severe compression of the exiting L5 nerve root, which was wedged between the disk space anteriorly and the S1 superior articulating process posteriorly.

The patient was returned to the operating room for L5 laminectomy and complete right L5-S1 facetectomy. Six months following surgery, the patient was noted to have trace weakness with his right ankle dorsiflexion. Otherwise, he was neurologically intact. He denied significant back or leg symptoms. He was not requiring any pain medication, and he had resumed all normal activities.

## 3. Discussion

Complications can be associated with either the ALIF or the posterior instrumentation portions of the procedure. Reported complications are diverse and range from vascular injuries, bowel and ureter injuries, postoperative bleeding requiring reoperation, postoperative paralytic ileus, sacral insufficiency fracture, pseudoarthrosis, and neurological injury [9–13]. A review article on iatrogenic neurologic deficits after lumbar spine surgery reported a 4.1% rate of new-onset neurologic injury after anterior or lateral lumbar fusion surgery [10]. Postoperative L5 radiculopathies after L5-S1 ALIF procedures have been described [14, 15]. The postoperative deficit has most commonly been attributed to a stretch neuropraxia from over distraction of the disk L5-S1 space.

One potential complication of the L5-S1 ALIF with posterior instrumentation is exacerbation of foraminal stenosis when the tulip heads of pedicle screws are reduced to the rod. To the authors' knowledge, this complication has not been reported and discussed. Numerous studies have described the powerful phenomenon of indirect decompression of the neural elements using lateral or anterior interbody cages [16–18]. The reports describe how interbody cages can increase disk height, foraminal height, and cross-sectional area of the spinal canal and foramina. However, it is important to recognize that placement of spinal hardware, in particular rod and screw constructs, can introduce antero- or retrolisthesis, resulting in neural compression. In this case, the L5 pedicle screw vertebral body was inadvertently reduced to the rod, producing retrolisthesis of L5 on S1. The L5 root was compressed between the L5-S1 annulus and the superior articulating facet of S1, most significantly in the medial aspect of the lumbosacral tunnel.

Avoidance of this complication starts with careful analysis of the preoperative CT and MRI studies. In this case, the preoperative MRI (Figure 2) demonstrated high-grade foraminal stenosis in the anterior-posterior plane. Therefore, avoidance of any new retrolisthesis at this level would be critical to avoid worsening nerve compression. Direct nerve decompression should also have been considered.

As with any ALIF case, cautious technique is required during the cage trialing and cage insertion processes. Oversizing the graft and aggressive malleting with the cage trial should be avoided. These maneuvers have been associated with L5 radiculopathy after L5-S1 ALIF [14, 15]. The surgeon should also account for the geometric shape of the interbody cage. A hyperlordotic graft may actually reduce the anterior-posterior diameter and cross-sectional area of the foramen. In these cases, a posterior decompression may be needed.

When the posterior screws are placed, the position of the tulip heads on the pedicle screws should be noted. We now pay close attention to the relative positions of the tulip heads when we obtain our X-rays or O-arm™ (Medtronic, Minneapolis, MN) imaging studies. The contour of the rod and the position of the tulip heads should be matched such that the tulip heads are not reduced to the rod, unless this is the desired effect. Reduction of anterolisthesis or introduction of retrolisthesis may result L5 nerve compression in the lumbosacral tunnel. Another strategy to avoid undesired reduction of screws is to use a commercially available product such as Bendini® (Nuvasive, San Diego, CA). This product uses computer-assisted technology to bend rods precisely to match the tulip head locations.

Finally, intraoperative imaging studies obtained after rod placement should be studied carefully for new spinal malalignment which could result in neural compression.

## 4. Conclusion

During lumbar fusion surgery, securing rods to pedicle screws can introduce spondylolisthesis. When no direct nerve decompression is performed, surgeons must carefully match the rod contour to the screw positions to avoid exacerbation of foraminal stenosis and neural compromise.

## Data Availability

The underlying supportive data used to support the findings of this study are included within the article.
